# Theoretical Design of a Two-Photon Fluorescent Probe for Nitric Oxide with Enhanced Emission Induced by Photoninduced Electron Transfer

**DOI:** 10.3390/s18051324

**Published:** 2018-04-25

**Authors:** Yujin Zhang, Jiancai Leng, Wei Hu

**Affiliations:** 1 School of Science, Qilu University of Technology (Shandong Academy of Sciences), Jinan 250353, China; zhangyujin312@163.com (Y.Z.); jiancaileng@qlu.edu.cn (J.L.); 2 Hefei National Laboratory for Physical Sciences at the Microscale, iChEM (Collaborative Innovation Center of Chemistry for Energy Materials), School of Chemistry and Materials Science, University of Science and Technology of China, Hefei 230026, China

**Keywords:** two-photon fluorescent, photoninduced electron transfer, density functional theory

## Abstract

In the present work, we systematically investigate the sensing abilities of two recently literature-reported two-photon fluorescent NO probes, i.e., the o-phenylenediamine derivative of Nile Red and the p-phenylenediamine derivative of coumarin. The recognition mechanisms of these probes are studied by using the molecular orbital classifying method, which demonstrates the photoinduced electron transfer process. In addition, we have designed two new probes by swapping receptor units present on fluorophores, i.e., the p-phenylenediamine derivative of Nile Red and the o-phenylenediamine derivative of coumarin. However, it illustrates that only the latter has ability to function as off-on typed fluorescent probe for NO. More importantly, calculations on the two-photon absorption properties of the probes demonstrate that both receptor derivatives of coumarin possess larger TPA cross-sections than Nile Red derivatives, which makes a better two photon fluorescent probe. Our theoretical investigations reveal that the underlying mechanism satisfactorily explain the experimental results, providing a theoretical basis on the structure-property relationships which is beneficial to developing new two-photon fluorescent probes for NO.

## 1. Introduction

Nitric oxide (NO), a widely existing signal transduction molecule in biological systems, plays an important role in various physiological and pathological processes [1,2,3]. It takes part in many living activities, such as neurotransmitters in the central nervous system and as a mediator in the immune system [4,5,6,7,8]. Studies have shown that the misregulation of NO is implicated with diverse diseases, like cardiac disorders, gastrointestinal distress, and neurodegeneration [9,10]. In order to investigate the role that NO plays in biological systems, it is important to detect the distribution of NO. In the past decades, developing applicable methods to monitor NO in living cells has become an attractive subject [11]. Among various approaches that have been employed to detect NO, fluorescent imaging technique is regarded as one of the most promising one in view of its simplicity, high sensitivity, and real-time detection [12,13,14,15,16]. Thus, the design and synthesis of fluorescent probes for NO has drawn great attention.

To date, many fluorescent probes that are capable of detecting NO changes in living cells have been developed [17,18,19] and one-photon fluorescent probe has been widely utilized. As is known, the one-photon fluorescence microscopy is easily interfered by photodamage and photobleaching due to the short wavelength excitation. Simultaneously, the penetration depth and the spatial resolution are relatively limited [20,21]. In contrast to the one-photon fluorescent probe, a two-photon fluorescent probe takes two photons with lower energy for the excitation can overcome the above shortcomings [22,23,24]. Therefore, the development of two-photon fluorescent probe has been of great interest in many fields at present.

In general, the sensing performance of a two-photon fluorescent probe depends crucially on the two major parts of a fluorescent probe, namely, the receptor and the fluorophore. The receptor of a probe serves as the reaction site of the analyte, and the fluorophore acts as a fluorescent signal reporter. It is noted that an effective fluorophore of two-photon fluorescent probe shows large two-photon response [25]. Except for the receptor and fluorophore, the recognitional mechanism is another factor that must be taken into account to design a fluorescent probe. A number of strategies, including intermolecular charge transfer (ICT), photoinduced electron transfer (PET), fluorescence resonance energy transfer (FRET), and through-bond energy transfer (TBET), have been adopted to the probe design [26,27,28,29]. Thereinto, PET has been extensively investigated and successfully applied to live-cell imaging of NO due to its induced fluorescence off-on transform [30,31].

Recently, Liu et al., synthesized a far-red emissive probe(renamed as Pro1 in this paper instead of the original name NRNO in the experimental paper), using Nile Red as the fluorophore and the o-phenylenediamine as the receptor, for NO detection and imaging [32]. Then, after considering the deficiencies of the o-phenylenediamine moiety with interference from some reactive carbonyl/oxygen/nitrogen species, they designed a new fluorescent probe (renamed as Pro2 in this paper instead of the original name NCNO in the experimental paper) with mono alkyl substituted p-phenylenediamine as the recognition group for NO [33]. Both Pro1 and Pro2 show fast fluorescence response toward NO with a low detection limit in the experiments. In particular, these two probes can be successfully applied in two-photon microscopy imaging for NO in living cells. In the present work, not only are the two experimental probes selected for the theoretical investigation, we have also designed two novel probes (named hereafter as Pro3 and Pro4) by employing the same fluorophores of Pro1 and Pro2. On basis of the systematic study on the one-photon absorption (OPA), one-photon emission (OPE) and two-photon absorption (TPA) properties of the molecules, their sensing performances are compared. More importantly, the recognition mechanisms of the probes are discussed by analyzing the molecular orbitals distribution diagrams. The effect of receptor and fluorophore on sensing performance of the probes is, thus, demonstrated. We hope the study can provide helpful information on the structure-property relationships for further investigating and designing two-photon fluorescent NO probes.

## 2. Theoretical Method and Computational Details

### 2.1. Theoretical Method

The transition probability for one photon absorption and emission can be specified by the oscillator strength:

(1) $$\delta_{OP}=\frac{2\omega_{ij}}{3}{\sum_{\alpha }\left|\langle i|\mu_{\alpha }|j\rangle \right|}^{2},$$

where ω*_ij_* denotes the excitation energy between the states *i* and *j*, *μ_α_* is the Cartesian component of the electronic dipole moment operator. The summation is performed over the *x*, *y*, and *z* axes.

The TPA cross-section is directly related to the imaginary part of the third order susceptibility of a molecule. Alternatively, it can be obtained by calculating the two-photon transition matrix elements *S_αβ_* between the initial state *i* and final state *f* [34,35]:

(2) $$S_{\alpha \beta }=\sum_{s}\left[\frac{\langle i|\mu_{\alpha }|s\rangle \langle s|\mu_{\beta }|f\rangle }{\omega_{si}-\omega }+\frac{\langle i|\mu_{\beta }|s\rangle \langle s|\mu_{\alpha }|f\rangle }{\omega_{si}-\omega }\right].$$

Here, *ω* represents the fundamental frequency of the incident light and 2*ω* = *ω_if_*, corresponding to a resonant TPA process. The summation here includes all initial, intermediate, and final states.

The molecular TPA cross section is dependent on the polarization of the incident beams. When excited by a linearly-polarized monochromatic beam, the orientationally averaged TPA cross-section is given by [36]:

(3) $$\delta_{TPA}=\sum_{\alpha ,\beta }\left[2S_{\alpha \alpha }S_{\beta \beta }^{*}+2S_{\alpha \beta }S_{\alpha \beta }^{*}+2S_{\alpha \beta }S_{\beta \alpha }^{*}\right].$$

Furthermore, the macroscopic TPA cross section directly comparable with the experiment is defined as [35,37]:

(4) $$\sigma_{TPA}=\frac{4\pi^{2}a_{0}^{5}\alpha }{15c}\frac{\omega^{2}g\left(\omega \right)}{\Gamma_{f}}\delta_{TPA},$$

where *a*_0_ is the Bohr radius, *c* denotes the speed of light and *α* is the fine structure constant. The level broadening of the final state Γ*_f_* is set to 0.1 eV [38], which corresponds to lifetime of a few femtoseconds.

### 2.2. Computational Detail

In order to choose suitable functional to optimize the molecular geometries and calculate their optical properties, the two experimental probes Pro1 and Pro2 were taken as examples to be calculated with different functionals. In addition to the common hybrid functional B3LYP, the CAM-B3LYP, M05-2X, M06-2X, PBE, and WB97XD functionals were also used for the test. Herein, all of the functionals were implemented with the 6-31G(d,p) basis set. It is worth noting that, during the experiments [32,33], the measurements were carried out in solution, so the influence of solvent was important to reproduce the experimental results with reasonable accuracy. To achieve this objective, the solvent effect of water was simulated using the polarizable continuum model (PCM) [39] in all computations.

The ground-state geometries of the molecules were fully optimized. No imaginary frequency was obtained, indicating that the equilibrium geometries were stable. On the basis of the optimized structures, the OPA properties of the molecules were calculated and the corresponding absorption wavelengths and oscillator strengths were shown in Table 1.

The results in Table 1 show that OPA maxima calculated by the B3LYP functional are in reasonable agreement with the experimental values. Thus, the B3LYP functional combined with the 6-31G(d,p) basis set was adopted to do all the calculations. The fluorescence properties were obtained by optimizing the first excited state (S1) geometry of the molecules. All of the above calculations were carried out in the Gaussian 09 (Gaussian, Inc., Wallingford, CT, USA) program package [40].

In addition, apart from investigating the OPA and OPE properties of the studied molecules, the other main task of this work is to explore their TPA features. Thus, the TPA cross-sections which can represent the TPA characteristics are evaluated by means of the quadratic response theory implemented in the Dalton2013 program [41].

## 3. Result and Discussion

### 3.1. Molecular Structure

**Figure 1:**
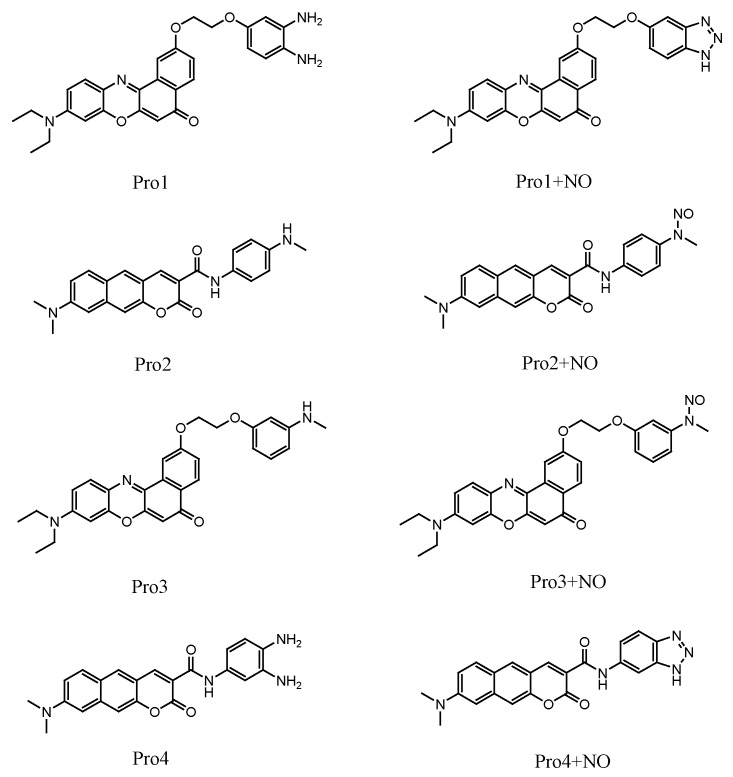
Molecular structures of the studied molecules.

A typical fluorescent probe contains a fluorophore (the fluorescent signal reporter) connected to a receptor (the recognition site of the analyte). As shown in Figure 1, the fluorophore Nile Red and coumarin derivate are connected with the reporter o-phenylenediamine and p-phenylenediamine, forming the probes Pro1 and Pro2, respectively. The rigid linkers of oxygen-containing carbon chain in Pro1 and amide bond in Pro2 can provide a suitable distance between the fluorophore and receptor to avoid the static fluorescence quenching. Based on the referenced molecule of Pro1 and Pro2, we have designed two probes (Pro3 and Pro4) by taking the same fluorophores and linkers of Pro1 and Pro2 accordingly and changing the receptors (see Figure 1).

Optimized ground state molecular geometries are presented in Figure S1. As is shown, there is large tortuosity between the probes’ fluorophore and receptor, namely, the probes are in noncoplanar conformations. Moreover, the presence of NO induces transformations in the receptors, whereas this leads to a slight change on the noncoplanar conformations of the molecules.

### 3.2. One-Photon Absorption Property

**Figure 2:**
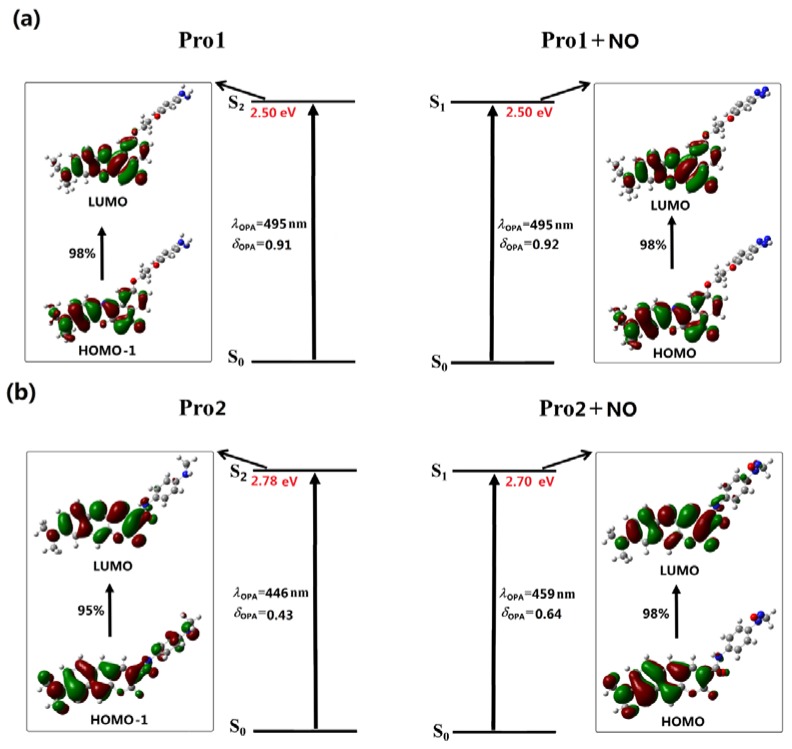
The OPA of (**a**) Pro1, Pro1 + NO and (**b**) Pro2, Pro2 + NO.

**Figure 3:**
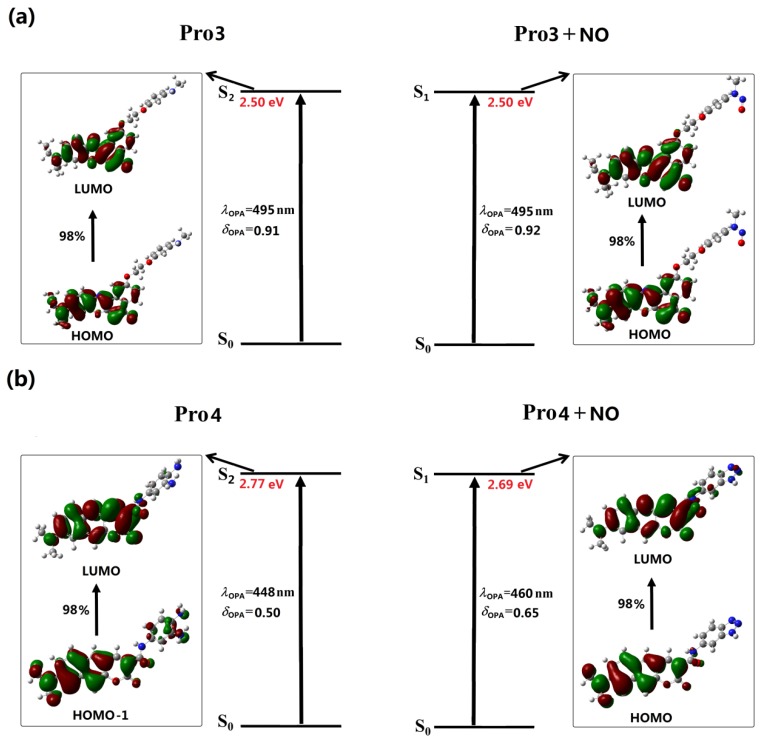
The OPA of (**a**) Pro3, Pro3 + NO and (**b**) Pro4, Pro4 + NO.

In the experiments, the maxima absorption wavelength of Pro1 and Pro2 remained nearly unchanged upon the addition of NO. To better explain this phenomenon, the OPA properties of all the studied molecules have been calculated based on the optimized geometries. The calculated results, including OPA wavelengths, the corresponding oscillator strengths and the transition natures are depicted in Figure 2 and Figure 3. The detailed and the reported experimental data are listed in Table S1.

In Figure 2, the absorption maxima of the chromophores Pro1 and Pro2 are located at 495 nm and 446 nm, respectively. With the presence of NO, the absorption peak of Pro1 + NO remains 495 nm while that of Pro2 + NO slightly redshifts to 459 nm, which is in reasonable agreement with the experimental observations. However, compared with the experimental values (583 and 585 nm for Pro1 and Pro1 + NO measured in PBS buffer containing 10% DMF as co-solvent, 473 and 475 nm for Pro2 and Pro2 + NO measured in PBS buffer containing 10% CH_3_CN as the co-solvent), the calculated values show redshifts, especially for Pro1 and Pro1 + NO. This discrepancy may results from several aspects: first, the solution environments are different for the experiments and the calculations; second, intermolecular interaction in the solution, including solvent-solute interaction and solute-solute interaction has not been considered in the calculation.

Careful inspects on the transition natures of Pro1 and Pro2 with the absence and presence of NO show that the maximum OPA states of both Pro1 and Pro2 are the second excited states, whereas those of Pro1 + NO and Pro2 + NO are the first excited states. In particular, the maximum OPA states of Pro1 and Pro2 are originated from the HOMO – 1 to LUMO transition (HOMO represents the highest occupied molecular orbital and LUMO represents the lowest unoccupied molecular orbital), and those of Pro1 + NO and Pro2 + NO are resulted from the HOMO to LUMO transition. Given that molecular orbitals involved in the absorption processes of the molecules are mainly localized on the fluorophore moiety, the absorption of Pro1 and Pro2 are mainly distributed there both in the absence and presence of NO.

With regard to Pro3 and Pro4, similar absorption wavelengths as Pro1 and Pro2 are respectively shown (see Figure 3), which is attributed to the fluorophore-localized transitions that mainly contribute to the absorption peaks. Although the receptor part for Pro1 and Pro3, as well as for Pro2 and Pro4 are different, the OPA properties of the molecules are similar. This indicates the OPA of these molecules is mainly contributed by the fluorophore, revealing that there is no strong electronic interaction between the molecular fluorophore and receptor.

### 3.3. One-Photon Emission Property

An efficient fluorescent probe exhibits observable change in its fluorescent signal after reacting with the analyte. To investigate the fluorescent properties of the studied molecules, the geometries in the first excited state S1 are optimized and shown in Figure S2. It can be seen that compared with the ground state geometries, the first excited state geometries have slight changes for Pro1 and Pro3. However, the torsion angles between the fluorophore and the receptor for Pro2 and Pro4 are obviously increased for the first excited state than the ground state. Upon excitation, the molecule reaches the excited state. Then, the subsequent fast and non-radiative internal conversion and vibrational relaxation processes lead the molecule to be located at the lowest vibrational level on the first excited state according to Kasha’s rule. During this process, the molecular configuration will change rapidly through the conformation transformation process.

**Figure 4:**
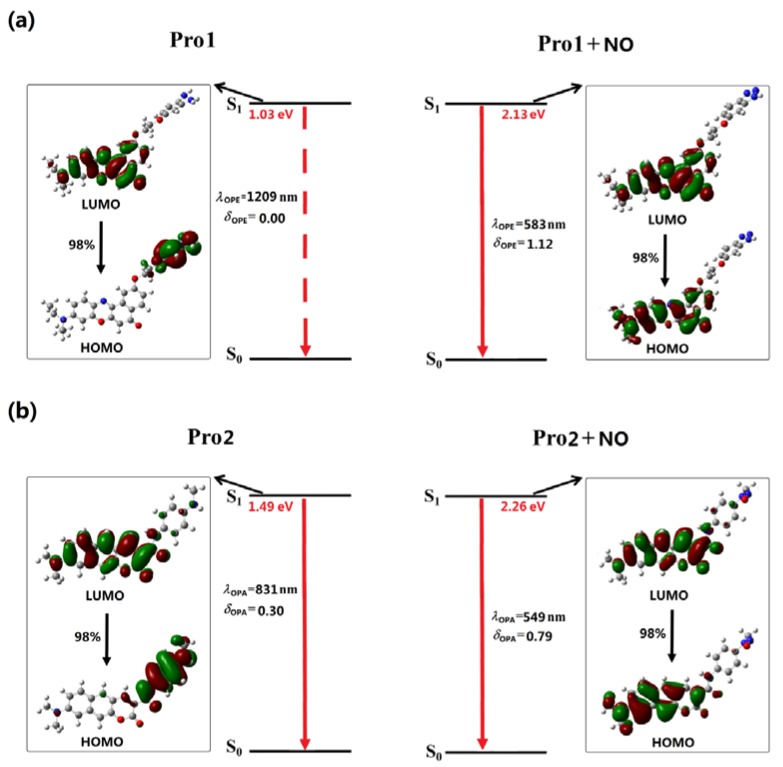
The OPE of (**a**) Pro1, Pro1 + NO and (**b**) Pro2, Pro2 + NO.

**Figure 5:**
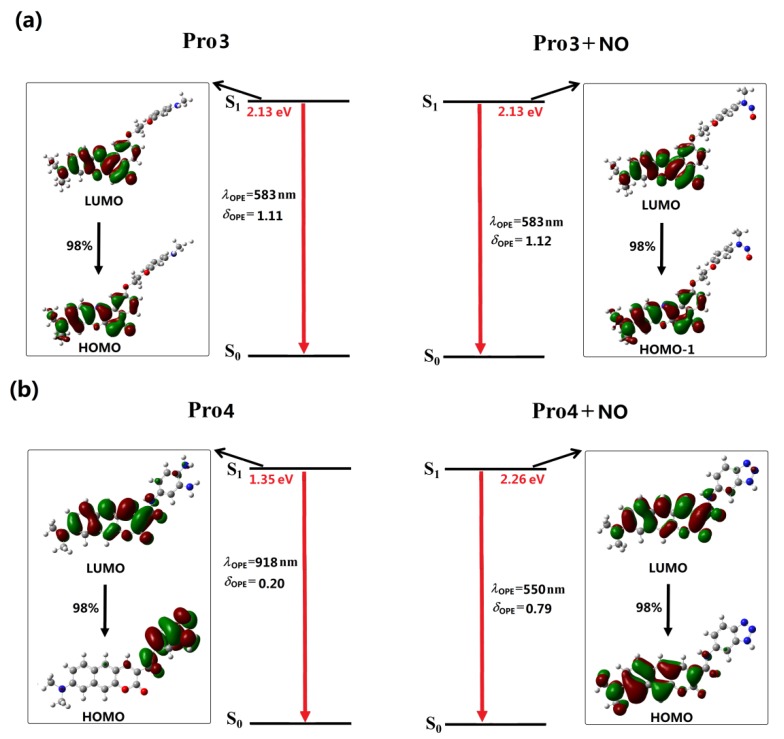
The OPE of (**a**) Pro3, Pro3 + NO and (**b**) Pro4, Pro4 + NO.

In Figure 4 and Figure 5, the OPE processes of the studied molecules are shown, and the detailed OPE wavelengths, the corresponding oscillator strengths, and transition natures are given in Table S2. It can be seen from Figure 4a that the S1 to S0 transition of Pro1 results from the LUMO to HOMO transition, denoting an electron transfer process from the fluorophore to the receptor moiety. The calculated oscillator strength for this transition is 0. Thus, the first excited state of Pro1 is a dark state, meaning that the direct S1 to S0 transition is forbidden and the molecule is non-fluorescent.

Figure 4a shows that the emission of Pro1 + NO originates from the LUMO to HOMO transition, which is localized on the molecular fluorophore moiety. As the transition probability between two molecular orbitals depends on their orbital distribution overlap [42], a greatly-enhanced emission intensity of Pro1 + NO can be achieved. Thus, when it reacts with NO, an emissive S1 state is observed for Pro1 + NO with a wavelength of 583 nm and an oscillator strength of 1.12. Considering the emission oscillator strength of Pro1 and Pro1 + NO, the fluorescence off-on switching ability of Pro1 for NO is rationalized, which is in good accordance with the experimental observations.

Although the molecular orbitals contributed to the emission of Pro2 is partly overlapped, leading to a small intensity of its emission (see Figure 4b), the OPE wavelength of 831 nm is beyond the range of visible light. Thus, the free Pro2 is also non-fluorescent. However, the addition of NO changes the emission of the probe. A fluorophore moiety localized S1 to S0 transition is observed for Pro2 + NO, thus, the molecule is emissive with a wavelength of 549 nm and an oscillator strength of 0.79. The fluorescence off-on effect is also shown in Pro2 for NO, finely according with the experiment [33]. It is worth noting that our calculations agree well with the experimental fluorescent wavelength trends whereas quantitative differences still exist, which might be contributed by the vibrational components of the emission that have not been considered in our calculation.

With the same fluorophore moiety of Pro1, the fluorescence of Pro3 is totally different (see Figure 5a). As the inverse process of OPA (see Figure 3), the OPE of Pro3, originating from the LUMO to HOMO transition, is mainly localized on the fluorophore. With the presence of NO, the transition nature of Pro3 + NO is similar as that of Pro3. In addition, the HOMO and LUMO distributions of Pro3 and Pro3 + NO are extremely alike. Thus, the emission wavelength and intensity of Pro3 before and after reacting with NO are almost unchanged, indicating that recognizing NO through the fluorescent variation cannot be achieved by Pro3.

In Figure 5b, the similar transition natures as Pro2 are shown in Pro4. Namely, the free probe exhibits an invisible emission wavelength with weak intensity, and when reacting with NO, a 550 nm fluorophore-localized fluorescence is greatly enhanced. Therefore, this probe can act as a promising candidate for the off-on typed fluorescent chemosensor.

As is well-known, a large Stokes shift can efficiently avoid the interference between the absorption and emission spectra, which is useful to improve the sensitivity and accuracy of the detection. In this study, the Stokes shift of the probes after reacting with NO is calculated. The results are 88 nm, 90 nm, 88 nm, and 90 nm for Pro1 + NO, Pro2 + NO, Pro3 + NO, and Pro4 + NO, respectively. This exhibits that molecules with coumarin derivate as the fluorophore have a larger Stokes shift, revealing the superiority of this fluorophore.

In general, analyses on the OPE properties of the molecules indicate that, except for Pro3, other molecules feature obvious off-on fluorescent response to NO, and Pro2 and Pro4 have more obvious Stokes shifts compared with Pro1. The influence of structure on the fluorescent properties is thus demonstrated, indicating the importance of choosing a suitable molecular fluorophore and receptor.

### 3.4. Recognition Mechanism

**Figure 6:**
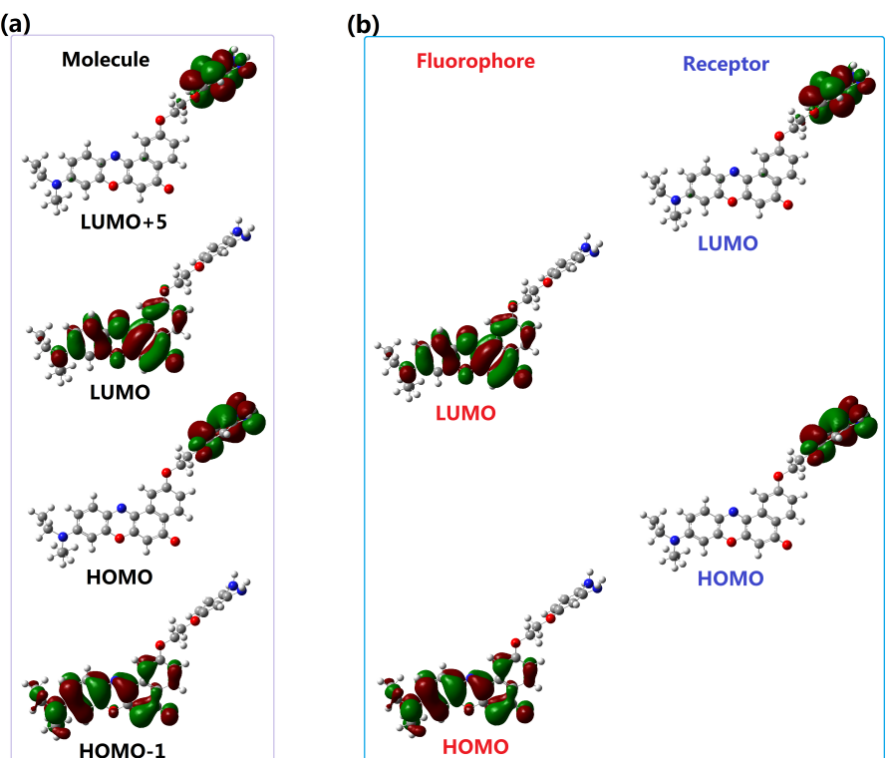
(**a**) The frontier molecular orbitals and (**b**) the separation of the orbitals for the fluorophore and receptor of Pro1.

**Figure 7:**
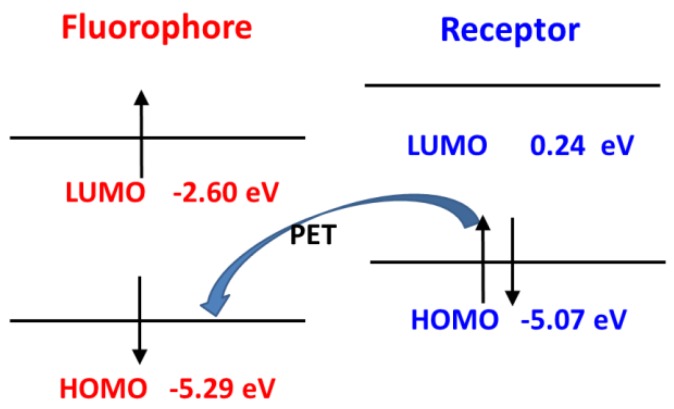
The PET process of Pro1.

As reported in the experiments [32,33], the recognition of NO for Pro1 and Pro2 pertains to the PET mechanism, However, theoretical elucidations on the mechanism for both the experimental and our designed probes have not been carried out to date. On the basis of the OPA and OPE properties of the molecules, we intend to further investigate the recognition mechanisms of the probes by using the molecular orbital distribution diagrams. Some frontier molecular orbitals of Pro1 are given in Figure 6a. One can see that a part of orbitals, such as HOMO − 1and LUMO, are mainly constrained on the fluorophore moiety, while others, including HOMO and LUMO + 5, are on the receptor part. Hereby, these molecular orbitals can be divided to the fluorophore’s orbitals and the receptor’s orbitals, respectively [43], as shown in Figure 6b. That is, the HOMO − 1 (and HOMO) and LUMO (LUMO + 5) of the whole molecule are, respectively, the HOMO and LUMO of the fluorophore (and receptor). The energies of the corresponding orbitals are labeled in Figure 7. It is clear that the energy of the receptor’s HOMO is −5.07 eV, and it is higher than the energy of the fluorophore’s HOMO of −5.29 eV. As a consequence, when excited by the light, the electron in the HOMO of the fluorophore is promoted to the LUMO of the fluorophore, following by the electron transfer from the HOMO of the receptor to that of the fluorophore, which causes fluorescence quenching of the fluorophore.

**Figure 8:**
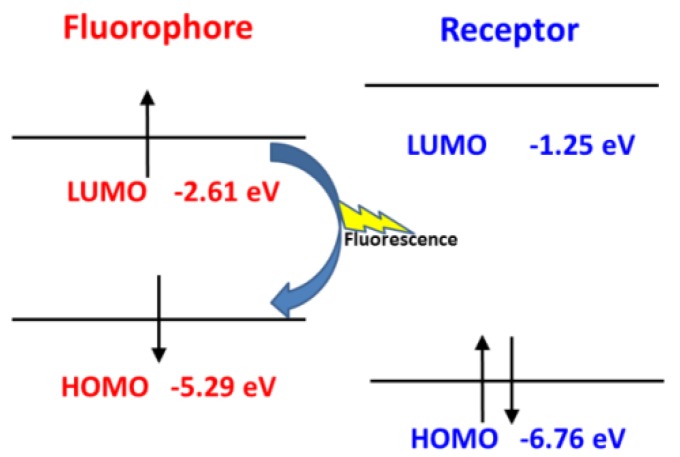
The inhibited PET process of Pro1 + NO.

However, after reacting with NO, the frontier molecular orbitals and the separation of the orbitals for the fluorophore and receptor of Pro1 + NO are shown in Figure S3. In Figure 8, one can see that, for Pro1 + NO, the energy of the HOMO for the receptor (−6.76 eV) is lower than the energy of the HOMO for the fluorophore (−5.29 eV). Thus, the electron only can transfer between the HOMO and the LUMO of the fluorophore, which inhibits the PET process and a significant fluorescence enhancement is achieved.

**Figure 9:**
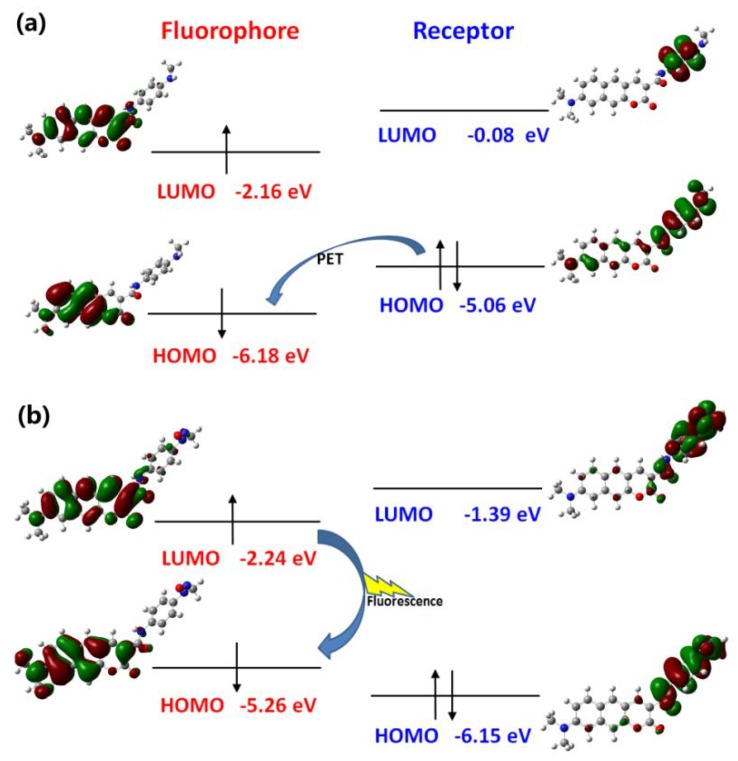
The PET and inhibited PET processes of (**a**) Pro2 and (**b**) Pro2 + NO.

In the case of Pro2, the mechanism can be illustrated in terms of molecular orbitals by Figure 9. According to the approach mentioned above, orbitals with distribution mainly located in the fluorophore and receptor part are picked out to form orbitals of the fluorophore and receptor, respectively. Then the HOMO − 1 (and HOMO) and LUMO (LUMO + 3) of the whole molecule Pro2 corresponds with respect to the HOMO and LUMO of the fluorophore (and receptor). This shows that the HOMO energy of the fluorophore (−5.06 eV) is higher than that of the receptor (−6.18 eV). Upon light excitation, the electron will transfer from the receptor to the fluorophore and the fluorescence is quenched, which induces the PET process. With the presence of NO, the HOMO energy of the receptor is lower than that of the fluorophore. Consequently, the PET process is inhibited and the emission of the fluorophore is recovered.

In the case of the designed probe, the PET and inhibited PET processes of Pro4 and Pro4 + NO are depicted in Figure S4, which resembles that of Pro2 as we have discussed above.

### 3.5. Two-Photon Absorption Property

It has been demonstrated that Pro1, Pro2, and Pro4 can effectively identify NO through the PET process. In order to further investigate the utility of these probes as two-photon fluorescent chemosensors, their TPA properties, including the excitation energy, the corresponding TPA wavelength, and the TPA cross-section of the lowest ten excited states are summarized in Table S3, and the data in the range of 700–900 nm are listed in Table 2. Here the TPA properties of Pro3 and Pro3 + NO are also calculated to make a comparison. From the data in Table 2, it can be seen that Pro1 and Pro3 do not have significant two-photon responses both in the absence and presence of NO (σ_TPA_ < 50 GM). However, the maximum TPA cross-section is slightly increased when the probes react with NO. For Pro2 and Pro4, the maximum TPA cross-sections are 171 GM and 116 GM, respectively, which increase to 183 GM and 173 GM upon the addition of NO. The trend of enhancement in the TPA cross-section of Pro1 and Pro2 with the presence of NO is consistent with the experimental measurements. Nevertheless, theoretical values are somewhat different from the experimental ones (see Table 2). The main reason for this is that the experimental values are the two-photon action cross-section, which is defined as the product of two-photon absorption cross-section and the fluorescence quantum yield.

As shown in Equation (3), TPA response can be estimated by the value of the two-photon transition matrix element. To interpret the origin of TPA properties of the studied molecules, the corresponding two-photon transition tensors are listed in Table 3. It is found that the larger two-photon transition tensor leads to an increased TPA cross-section, suggesting the transition dipole moments and frequency detunings between the incident light and the excitation energy are critical factors influencing the value of the TPA cross-section.

Importantly, the maximum TPA cross-sections of Pro2 and Pro4 are about four times larger than those of Pro1 and Pro3, which can be ascribed to the fluorophore. One can thus predict that the coumarin derivate is a preferable fluorophore than Nile Red in a two-photon fluorescent probe. Although both Pro2 and Pro4 exhibit enhancement on the TPA cross-section when reacted with NO, the increment of Pro4 (57 GM) is much larger than Pro2 (12 GM), showing the influence of the receptor on the TPA properties of the probes. The above result confirms the superiority of the designed probe Pro4 on two-photon microscopy imaging for NO in living cells.

When excited by a source with a wavelength of 869 nm, Pro4 will be elevated to the excited state. However, no fluorescence is emitted because of the PET-induced fluorescence quenching. With the presence of NO, the PET process is impeded in Pro4 + NO and strong fluorescence from the fluorophore can be observed after the excitation of incident light. As a result, the identification of NO is achieved by Pro4.

## 4. Conclusions

In summary, we theoretically investigate the optical performances of two newly-synthesized and two designed two-photon fluorescent NO probes. The OPA property studies indicate that the OPA of the molecules change slightly after reacting with NO, which agrees with the experimental measurements. Especially, calculations on the emission property of the molecules suggest that, except for Pro3, the fluorescence of other molecules is quenching and can be turned on by the addition of NO. It shows that, apart from Pro3, the other molecules exhibit obvious fluorescence-off to fluorescence-on transformation in the presence of NO, indicating them to be good fluorescent chemosensors for NO. In order to further investigate the recognition mechanism of the probes, the molecular orbital classifying method is used and the PET process is theoretically demonstrated. More importantly, the novel probe Pro4 has a larger TPA cross-section than other probes. Therefore, it is deduced that the coumarin derivate can act as a promising fluorophore in a two-photon fluorescent probe and Pro4 possesses great potential to be an excellent two-photon fluorescent chemosensor for NO. Our calculated results reveal in-depth insight into the NO probes with good TPA activity, providing a theoretical basis on the structure-property relationships which is beneficial to synthesizing more PET-based NO-responsive two-photon fluorescent sensors.

## Figures and Tables

**Table 1:** The maximum OPA wavelength *λ_OPA_* (nm) and oscillator strength δ*_OPA_* (a.u.) of Pro1, Pro1 + NO, Pro2, and Pro2 + NO with different functionals.

Functionals	Pro1		Pro1 + NO		Pro2		Pro2 + NO	
	*λ_OPA_*	δ*_OPA_*	*λ_OPA_*	δ*_OPA_*	*λ_OPA_*	δ*_OPA_*	*λ_OPA_*	δ*_OPA_*
B3LYP	495	0.91	495	0.92	446	0.43	459	0.64
CAM-B3LYP	451	1.05	451	1.06	380	0.93	383	0.91
M05-2X	451	1.09	451	1.09	381	0.98	383	0.95
M06-2X	451	1.07	451	1.08	384	0.96	387	0.94
PBE	485	0.96	485	0.96	428	0.39	442	0.70
WB97XD	449	1.05	449	1.06	373	0.92	376	0.90
Experiment	583	-	585	-	473	-	475	-

**Table 2:** The two-photon absorption energy E_TPA_ (eV), the corresponding TPA wavelength λ_TPA_ (nm), TPA cross-section σ_TPA_ (GM = 10^−50^ cm^4^·s/photon) for the studied molecules in the range of 700–900 nm.

Molecule	*E_TPA_*	*λ_TPA_*	*σ_TPA_*	Molecule	*E_TPA_*	*λ_TPA_*	*σ_TPA_*
Pro1	2.98 3.02 3.09 3.39	832 818 801 731	0 25 7 13	Pro1 + NO	3.00 3.09 3.30 3.39	826 801 750 730	0 29 8 10 38 *
Pro2	2.77 3.42	892 724	171 6 2.4 *	Pro2 + NO	3.26 3.36 3.44	758 736 719	3 183 0 54 *
Pro3	2.98 3.09 3.18 3.39	829 801 779 731	0 28 6 13	Pro3 + NO	2.99 3.09 3.29 3.35 3.39	826 801 753 740 730	0 29 0 1 13
Pro4	2.76 3.39 3.43	896 729 722	116 56 6	Pro4 + NO	3.36 3.51	737 706	173 42

* Two-photon action cross-section measured in the experiments [32,33].

**Table 3:** The two-photon transition tensors (a.u.) for the studied molecules.

Molecule	S_xx_	S_yy_	S_zz_	S_xy_	S_xz_	S_yz_
Pro1	4.4	16.6	127.3	−1.8	−22.5	−12.5
Pro1 + NO	−5.0	−19.4	−133.8	1.9	22.7	17.9
Pro2	0.8	−4.6	−354.9	−2.1	1.9	43.8
Pro2 + NO	−0.5	−1.7	−340.8	−0.9	−18.5	−11.6
Pro3	3.5	17.5	133.6	−1.6	−20.8	−12.7
Pro3 + NO	3.1	18.6	136.5	−2.1	−18.1	−14.4
Pro4	1.0	−0.4	−294.7	−0.7	−1.4	36.3
Pro4 + NO	0.3	1.6	329.1	−0.8	15.4	16.3

## Supplementary Materials

The following are available online at http://www.mdpi.com/1424-8220/18/5/1324/s1.
