# Supplementary material for: Theoretical Design of a Two-Photon Fluorescent Probe for Nitric Oxide with Enhanced Emission Induced by Photoninduced Electron Transfer
Source: Sensors (Basel). 2018 Apr 25;18(5):1324. doi: 10.3390/s18051324 (PMC5982152; doi:10.3390/s18051324)
Supplement: Supplementary file 1 [file sensors-18-01324-s001.pdf]

Supporting Information for

Theoretical Design of a Two-photon Fluorescent Probe for Nitric Oxide with Enhanced Emission Induced by Photoninduced Electron Transfer

Yujin Zhang [1,†], Jiancai Leng [1,†] and Wei Hu [2,*]

[1] School of Science, Qilu University of Technology (Shandong Academy of Sciences), Jinan 250353, China; zhangyujin312@163.com (Y.Z.); jiancaileng@qlu.edu.cn (J.L.)

[2] Hefei National Laboratory for Physical Sciences at the Microscale, iChEM (Collaborative Innovation Center of Chemistry for Energy Materials), School of Chemistry and Materials Science, University of Science and Technology of China, Hefei 230026, China

* Correspondence: weihulp@ustc.edu.cn.

† These authors contribute equally to this work.

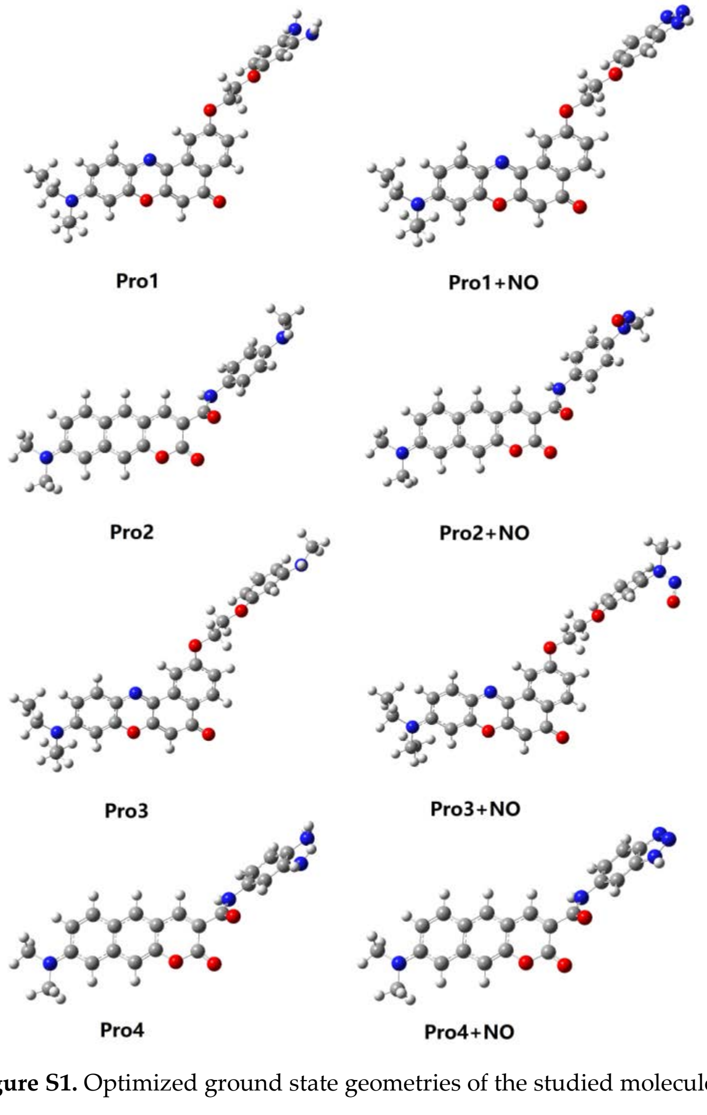

Figure S1. Optimized ground state geometries of the studied molecules.

Table S1. The OPA energy E_{OPA} (eV), the corresponding wavelength λ_{OPA} (nm), oscillator strength δ_{OPA} (a.u.) and the transition nature of the studied molecules.

Molecule	Excited State	E_{OPA}	λ_{OPA}	δ_{OPA}	Transition Nature	Exp/nm
Pro1	S2	2.5039	495.16	0.9134	HOMO-1-LUMO 98%	583
Pro1 + NO	S1	2.5034	495.26	0.9183	HOMO-LUMO 98%	585
Pro2	S2	2.7788	446.17	0.4258	HOMO-1-LUMO 95%	473
Pro2 + NO	S1	2.7009	459.04	0.6440	HOMO-LUMO 98%	475
Pro3	S2	2.5038	495.18	0.9111	HOMO-LUMO 98%	---
Pro3 + NO	S1	2.5035	495.24	0.9143	HOMO-LUMO 98%	---
Pro4	S2	2.7666	448.14	0.5011	HOMO-1-LUMO 98%	---
Pro4 + NO	S1	2.6941	460.21	0.6478	HOMO-LUMO 98%	---

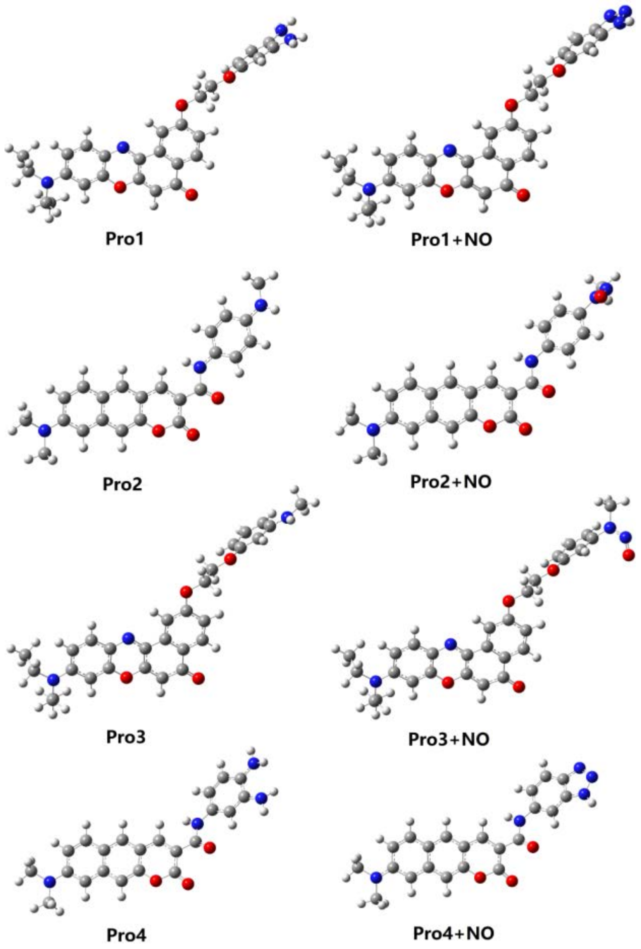

Figure S2. Optimized first excited state geometries of the studied molecules.

Table S2. The OPE energy E_{OPE} (eV), the corresponding wavelength λ_{OPE} (nm), oscillator strength δ_{OPE} (a.u.) and the transition nature of the studied molecules.

Molecule	Excited State	E_{OPE}	λ_{OPE}	δ_{OPE}	Transition Nature	Exp/nm
Pro1	S1	1.0254	1209.08	0.0002	LUMO-HOMO 98%	648

Pro1 + NO	S1	2.1261	583.16	1.1212	LUMO-HOMO 98%	650
Pro2	S1	1.4924	830.77	0.2983	LUMO-HOMO 98%	608
Pro2 + NO	S1	2.2577	549.16	0.7924	LUMO-HOMO 98%	613
Pro3	S1	2.1251	583.42	1.1185	LUMO-HOMO 98%	---
Pro3 + NO	S1	2.1256	583.30	1.1204	LUMO-HOMO 98%	---
Pro4	S1	1.3502	918.24	0.2005	LUMO-HOMO 98%	---
Pro4 + NO	S1	2.2552	549.76	0.7906	LUMO-HOMO 98%	---

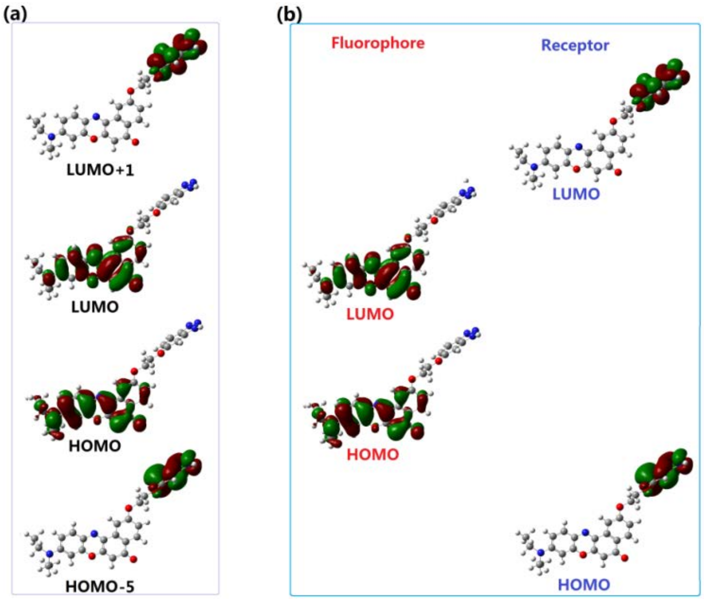

Figure S3. (a) The frontier molecular orbitals and (b) the separation of the orbitals for the fluorophore and receptor of Pro1 + NO.

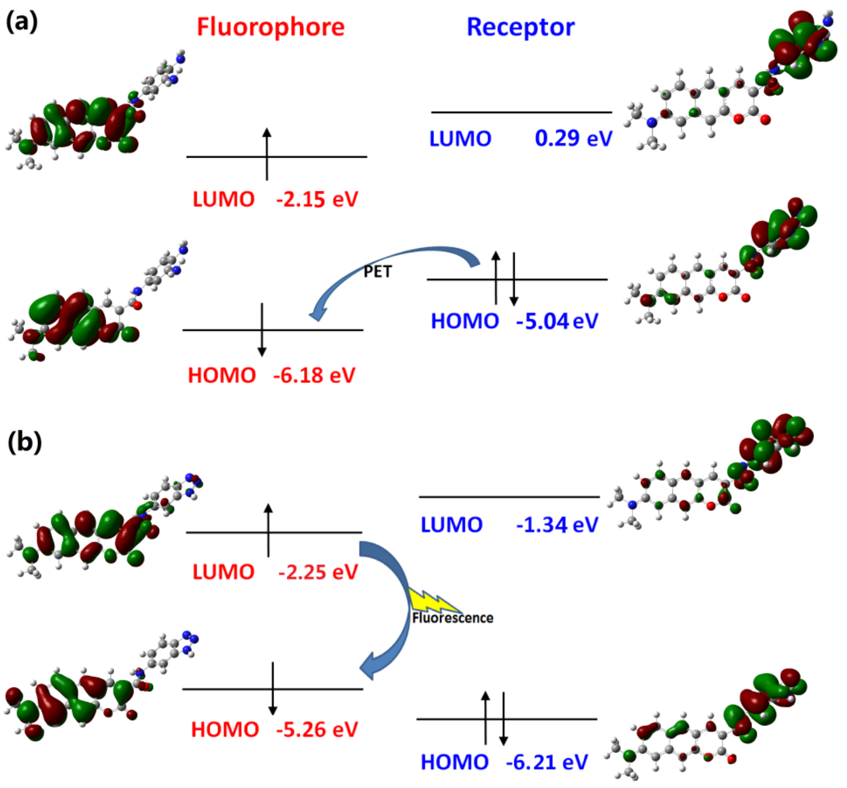

Figure S4. The PET and inhibited PET processes of (a) Pro4 and (b) Pro4 + NO.

Table S3. The TPA energy E_{TPA} (eV), the corresponding wavelength λ_{TPA} (nm) and the TPA cross section σ_{TPA} (GM) for the lowest ten excited states of the studied molecules.

Molecule	E_{TPA}	λ_{TPA}	σ_{TPA}	Molecule	E_{TPA}	λ_{TPA}	σ_{TPA}
Pro1	2.2061	1124.04	1	Pro1 + NO	2.5034	990.52	30
	2.5039	990.32	26		3.0003	826.48	0
	2.9803	832.02	0		3.0922	801.92	29
	3.0283	818.84	25		3.3029	750.76	8
	3.0921	801.94	7		3.3968	730	10
	3.3900	731.46	13		3.8068	651.38	0
	3.7574	659.94	0		3.8123	650.44	1
	3.8080	651.18	2		3.8237	648.5	79
	3.8212	648.92	182		3.8896	637.52	2
	3.9427	628.92	47		3.9488	627.96	140
Pro2	2.4958	993.54	212	Pro2 + NO	2.7009	918.08	84
	2.7788	892.34	171		3.2698	758.36	3
	3.4232	724.38	6		3.3667	736.54	183
	3.8152	649.94	21		3.4482	719.12	0
	3.9582	626.46	8		3.5962	689.52	26
	4.0325	614.92	68		3.8275	647.86	11
	4.2666	581.18	236		3.8831	638.58	197
	4.3065	575.8	14		4.0826	607.38	143
	4.3948	564.24	255		4.2371	585.22	46
	4.4923	551.98	329		4.3773	566.48	10
Pro3	2.4957	993.56	0	Pro3 + NO	2.5035	990.48	29

	2.5038	990.36	27		2.9997	826.66	0
	2.9898	829.38	0		3.0924	801.86	29
	3.0925	801.84	28		3.2917	753.32	0
	3.1828	779.08	6		3.3507	740.04	1
	3.3919	731.06	13		3.3965	730.08	13
	3.8077	651.22	0		3.6564	678.18	4
	3.8204	649.06	165		3.7506	661.16	0
	3.9451	628.56	61		3.8074	651.28	112
	4.0507	612.16	0		3.8187	649.34	7
Pro4	2.4770	1001.06	134	Pro4 + NO	2.6941	920.42	88
	2.7666	896.28	116		3.3637	737.18	173
	3.3976	729.84	56		3.5114	706.18	42
	3.4341	722.08	6		3.6084	687.18	5
	3.8102	650.8	3		3.8981	636.14	4
	3.9252	631.74	24		4.0717	609.02	200
	4.0546	611.56	76		4.0879	606.6	61
	4.2492	583.56	185		4.3760	566.66	85
	4.4534	556.8	32		4.5093	549.9	135
	4.4889	649.34	556		4.5319	547.16	6
